# 48 Iatrogenic Vitamin A Toxicity Leading to Hypervitaminosis and Hypercalcemia: A Case Series and Cautionary Tale

**DOI:** 10.1093/jbcr/irad045.022

**Published:** 2023-05-15

**Authors:** Alexandria Hall, Jessie King, Laura Tobias, Barclay Stewart, Lori Rhodes

**Affiliations:** Harborview Medical Center, Seattle, Washington; Harborview Medical Center, Seattle, Washington; Harborview Medical Center, Seattle, Washington; University of Washington, Seattle, Washington; Harborview Medical Center, Seattle, Washington; Harborview Medical Center, Seattle, Washington; Harborview Medical Center, Seattle, Washington; Harborview Medical Center, Seattle, Washington; University of Washington, Seattle, Washington; Harborview Medical Center, Seattle, Washington; Harborview Medical Center, Seattle, Washington; Harborview Medical Center, Seattle, Washington; Harborview Medical Center, Seattle, Washington; University of Washington, Seattle, Washington; Harborview Medical Center, Seattle, Washington; Harborview Medical Center, Seattle, Washington; Harborview Medical Center, Seattle, Washington; Harborview Medical Center, Seattle, Washington; University of Washington, Seattle, Washington; Harborview Medical Center, Seattle, Washington; Harborview Medical Center, Seattle, Washington; Harborview Medical Center, Seattle, Washington; Harborview Medical Center, Seattle, Washington; University of Washington, Seattle, Washington; Harborview Medical Center, Seattle, Washington

## Abstract

**Introduction:**

Patients with major burn injuries receive micro-nutrient supplementation to promote wound healing and mitigate hypermetabolic complications. Vitamin A promotes epithelialization, angiogenesis and collagen synthesis and is included in ‘trauma vitamin’ bundles. As a fat-soluble vitamin, prolonged supplementation can lead to cumulative toxicity. Hypercalcemia can cause polyuria, ECG changes, and organ dysfunction. We report a case series of potential iatrogenic vitamin A toxicity among people with major burn injury.

**Methods:**

Concern for vitamin A toxicity was raised by pharmacy and endocrinology after two patients were found with severe hypercalcemia and a consistent toxidrome. In response, we performed a chart review of adult patients with ≥20% TBSA who received vitamin A 25,000 units thrice weekly for at >2 weeks during March 2021 – July 2022. Hypercalcemia was defined as serum calcium concentration >10.4 mg/dL ( >2.60 mmol/L) or ionized serum calcium >5.2 mg/dL ( >1.30 mmol/L). Kruskal-Wallis test examined differences in median values.

**Results:**

A total of 67 patients met screening criteria with 25 included in the series. Nine patients (36%) developed hypercalcemia. One patient developed hypercalcemia on day 5 of admission and was excluded. Median days to hypercalcemia was 52 (IQR 39-72). There were no differences in age, burn size, or weight between patients with and without hypercalcemia. Patients with hypercalcemia had significantly longer courses of vitamin A (56 vs 27 days, p=0.03) as well as length of stay (122 vs 63 days, p=0.01). One patient required treatment with a bisphosphonate. All improved with cessation of vitamin A supplementation.

**Conclusions:**

A third of patients who received high-dose vitamin A as per a micronutrient supplementation protocol developed hypercalcemia. Our protocol was changed to limit vitamin A therapy to 21 days. Given that hypercalcemia among hospitalized patients is multifactorial, further evaluation with a larger case-control study is required to determine if high-dose vitamin A is a contributing factor.

**Applicability of Research to Practice:**

Recommended daily allowance of vitamin A is ~3,000 units daily. Given that patients on enteral feedings receive approximately 3,600 units daily, vitamin A supplementation may result in toxicity if provided for >3-5 weeks.

